# Implications of Tourist–Macaque Interactions for Disease Transmission

**DOI:** 10.1007/s10393-017-1284-3

**Published:** 2017-11-17

**Authors:** Charlotte Carne, Stuart Semple, Ann MacLarnon, Bonaventura Majolo, Laëtitia Maréchal

**Affiliations:** 1 Department of Life Sciences, University of Roehampton, London, UK; 2 School of Psychology, University of Lincoln, Sarah Swift Building, Brayford Wharf East, Lincoln, LN5 7AY UK

**Keywords:** Disease transmission risks, *Macaca sylvanus*, Modelling, Primates, Tourist–wildlife interactions, Wildlife tourism

## Abstract

During wildlife tourism, proximity or actual contact between people and animals may lead to a significant risk of anthropozoonotic disease transmission. In this paper, we use social network analysis, disease simulation modelling and data on animal health and behaviour to investigate such risks at a site in Morocco, where tourists come to see wild Barbary macaques (*Macaca sylvanus*). Measures of individual macaques’ network centrality—an index of the strength and distribution of their social relationships and thus potentially their ability to spread disease—did not show clear and consistent relationships with their time spent in close proximity to, or rate of interacting with, tourists. Disease simulation modelling indicated that while higher-ranked animals had a significantly greater ability to spread disease within the group, in absolute terms there was little difference in the size of outbreaks that different individuals were predicted to cause. We observed a high rate of physical contact and close proximity between humans and macaques, including during three periods when the macaques were coughing and sneezing heavily, highlighting the potential risk of disease transmission. We recommend that general disease prevention strategies, such as those aimed at reducing opportunities for contact between tourists and macaques, should be adopted.

## Introduction

As wildlife tourism continues to rise in popularity, contacts between humans and wild animals will inevitably increase (Chapman et al. 2009). This is likely to lead, in turn, to a greater risk of disease transmission for both tourists and the animals they have travelled to see (Wallis and Lee 1999; Daszak et al. 2000). Physical interactions with, or close proximity to, infected individuals significantly increase the risks of disease transmission, as pathogens can be transferred easily by touch, and sneezes can project infected droplets up to 12 m (Bourouiba et al. 2014). New and emerging zoonotic diseases have caused high mortality in human populations over recent years, with HIV and Ebola perhaps the best-known examples (Hahn et al. 2000; WHO 2014). Many diseases continue to spill over from wildlife populations to humans, often in a tourism context, for example African tick bite fever during wild game safaris (Ericsson et al. 2004) and rabies from monkey populations (Gautret and Parola 2012). A recent analysis showed that over 70% of new diseases that emerged between 1940 and 2004 originated in wildlife (Jones et al. 2008). There is also mounting evidence of zooanthroponosis, diseases transmitted from humans to animals, leading to significant mortality in populations of wild animals (Kaur et al. 2008).

Evidence for the introduction of diseases from humans to wild animals has been documented across a range of vertebrate taxa, with non-human primates appearing to be particularly vulnerable in this regard (Muehlenbein and Wallis 2014). Respiratory epidemics among great apes have been observed at a number of study sites (Cranfield 2008; Williams et al. 2008), and these outbreaks may have been caused by transmission from humans (Kaur et al. 2008; Koendgen et al. 2008). Tourists are often observed very close to primates and/or physically interacting with these animals by feeding or aggressing them (Sabbatini et al. 2006; Maréchal et al. 2011), which increases the risk of disease transmission from people to primates. In addition, tourists pose a particular risk to animals, as many are international visitors and thus more likely to introduce new pathogens to which animals do not have immunity (Muehlenbein and Wallis 2014). Such introduction of novel pathogens into fragile populations may have serious consequences for the survival of endangered species (Koendgen et al. 2008). In response to this risk, best practice guidelines have been produced by the IUCN in relation to great ape tourism (Macfie and Williamson 2010), and the recommended regulations have been introduced at a number of such tourism sites (Macfie and Williamson 2010).

With the exception of rabies, there are only a few definitive cases where there is evidence that a pathogen has spread from a primate to an individual person. For example, a human case of simian foamy virus was linked to a population of long-tailed macaques at a temple in Bali (Jones-Engel et al. 2005). Despite this evidence of potential disease transmission between humans and primates (Jones-Engel et al. 2005; Kaur et al. 2008; Koendgen et al. 2008), to date there is no documented case of transmission events between tourists and wild primates. Such events would be hard to identify, due to the transient nature of most wildlife tourism and a lack of longer-term monitoring of tourists after their visit, and also because of the difficulty of determining the origin of disease infections in wild animal populations (Muehlenbein and Wallis 2014). While studying actual disease transmission in wildlife tourism settings is extremely difficult, mathematical modelling approaches such as epidemiological models provide powerful tools to explore the risks of such transmission. Social network analysis has been used particularly in this regard, as there is increasing evidence that holding a central position in a social network is associated with higher infection risk (Drewe 2010), and may also be associated with a higher potential to spread diseases (Croft et al. 2008). Social network approaches have to date been used to facilitate the identification of the major potential routes of infection (Drewe 2010) and to examine how the structure of a social group can affect the potential spread of disease (Griffin and Nunn 2012; Rushmore et al. 2014). In this way, modelling studies can inform the development of guidelines related to wildlife tourism, reducing the threats to both people and animals.

In this paper, we investigate the factors that may increase the risks of disease transmission between tourists and wild adult Barbary macaques at a tourist site in Morocco. The macaque group we studied was visited daily by tourists, who often interacted with the animals by feeding or touching them. To understand the possible risks and impacts of a disease spreading from a tourist to the macaques, we investigated the potential for individual macaques to become infected, and once infected, the potential for them to spread disease within their social group. We first quantified how often each individual macaque interacted with, or was in close proximity to, tourists, as a measure of these animals’ exposure to potential disease transmission from people. We then tested whether animals’ dominance rank, sex or level of social integration were related to their time in proximity to, or rate of interaction with, tourists. Next, we simulated the spread of an infectious agent on observed social networks to assess the ability of each individual macaque to spread disease to other macaques if infected, depending on their rank, sex, proximity to, or rate of interactions with, tourists. Individuals that have a disproportionate ability to spread disease within their social group (i.e. ‘super-spreaders’) can potentially be effective targets for preventative measures (e.g. vaccination) to reduce the risk of disease outbreaks (Rushmore et al. 2013).

Modelling approaches to explore disease transmission risk are generally based on association networks collected during healthy periods, i.e. not while an outbreak is spreading, or animals have become ill. A potential criticism is that animals may alter their behaviour (including towards tourists) when infected, leading to considerable changes to the association network (Craft and Caillaud 2011; Lopes et al. 2016). Indeed, many infections are linked to increased levels of association in their hosts, facilitating transmission (Klein 2003; Moore 2013), while in some species individuals may actively avoid infected conspecifics (Kiesecker et al. 1999). Animals may also lower their activity levels when infected (Hart 1988; Nunn et al. 2015). Therefore, to test the validity of using the modelling approach, we also assessed the extent to which infection may have affected the social structure of the group.

As Barbary macaques were classified as endangered in 2008 (Butynski et al. 2008), and primate tourism has been proposed as a tool for their conservation (HCEFLCD 2012), it is particularly important to assess the associated potential disease transmission risks. In addressing this aim, the current study is the first to use modelling approaches informed by real-world data on interactions between tourists and primates.

## Methods

### Study Animals and Site

This study was conducted in Ifrane National Park in the Middle Atlas Mountains, Morocco (33°25′N; 005°10′W), on a group of Barbary macaques experiencing high tourism pressure every day (Maréchal et al. 2011). The study group was composed of 40 individuals at the start of the data collection: 12 adult males, 12 adult females, 2 sub-adult males, 1 sub-adult female, 6 juveniles and 7 one-year-old infants; 5 infants were born during the study period (Maréchal et al. 2016). Data were collected on 8 adult males and 9 adult females; young adults, sub-adults, juveniles and infants were excluded from the data collection, and two adults were excluded from the data collection as they disappeared or died at the start of the study period. The number of tourists at the site was highly variable between and within days, ranging from a solitary tourist to groups of over 100 people. The majority of tourists approached the macaques to within a few metres, and many interacted with them directly, giving them food or attempting to touch them.

### Data Collection

Data were collected between February and December 2012. Scan samples were taken every hour, a total of 10 scans per day, on all study animals that were visible. The nearest neighbour of each of the macaques and the approximate distance between them were recorded. Proximity to the nearest tourist group—defined as an aggregation of tourists within 3 m of each other—was also recorded. In addition, every 30 min, scans were taken to record the occurrence (or not) of any interactions macaques had with tourists which may present a risk of disease transmission, i.e. for each animal it was recorded that they were involved in a ‘feeding interaction’, ‘agonistic interaction’ or ‘no interaction’. Feeding interactions were defined as tourists giving food to macaques by hand, or by throwing it towards them. Agonistic interactions were defined as tourists threatening the macaques by throwing an object or making aggressive displays towards them, or attempting to contact them physically. Finally, any signs of respiratory illness (e.g. coughing or sneezing observed on several occasions during a day) exhibited by each macaque were recorded daily.

### Association Network Construction

An association network was created for the whole study period, based on nearest-neighbour associations within 10 m. This threshold distance was chosen as it is a common limit on human approach employed at great ape tourism sites, reflecting the estimated maximum distance over which respiratory infection transmission can occur (Homsy 1999; Nakamura and Nishida 2009). An association network represents the relationships within a social group or population. Individuals are represented by nodes, and if two individuals have been observed to associate, their respective nodes are connected by an edge (Croft et al. 2008). Networks can be either binary or weighted; in binary networks, relationships (edges) are either present or absent, while in weighted networks the strength of relationships is also included (Whitehead 2008). Here, edges were weighted using the dyadic association index:

$$ {\text{DAI}} = AB/\left( {A + B + AB} \right) $$

where *A* is the total number of times that *A* was observed without *B*, *B* is the total number of times that *B* was observed without *A* and *AB* is the total number of times that *A* and *B* were observed to be nearest neighbours within the given distance. This is equivalent to the simple ratio index (Cairns and Schwager 1987). Dyadic association indices range from zero to one, with zero indicating that two individuals were never observed to be nearest neighbours and one indicating that they were always observed as nearest neighbours. The network was not filtered, as we used a weighted network which minimises the impact of observation errors. Although this means that chance encounters are included in the network, this is justified in studies of disease transmission in which even chance occurrences provide an opportunity for disease spread (Croft et al. 2008).

The association network was calculated based on data from the whole study period. To assess the stability of the network over time, seasonal networks were also created for each of the seasons (winter 1 = February 2012; spring = March–May 2012; summer = June–August 2012; autumn = September–November 2012; winter 2 = December 2012) and the Quadratic Assignment Procedure in UCINET (Borgatti et al. 2002) was used to calculate correlations between each seasonal network and the overall network. In addition, to test whether the nearest-neighbour data over a 10 m distance was an accurate representation of overall social structure, we calculated the correlation between this network and one based on nearest-neighbour records within 1 m, using the Quadratic Assignment Procedure in UCINET (Borgatti et al. 2002).

### Network Analysis

Centrality in the network was quantified using weighted degree, weighted betweenness and weighted eigenvector centrality. Weighted degree centrality is a measure of the number and strength of each animal’s connections within the network (Croft et al. 2008). Weighted betweenness centrality is a measure of the number of weighted shortest paths on which a node lies; individuals with high betweenness centrality often connect groups of individuals that would otherwise be isolated, which can be particularly important for disease transmission (Wey et al. 2008). Weighted eigenvector centrality incorporates both the strength of an animal’s connections and the strength of connections held by that animal’s neighbours; an individual with high weighted eigenvector centrality is strongly connected to a lot of nodes that also have a lot of strong connections (Bonacich 1987). These network measures were calculated using igraph (Csardi and Nepusz 2006) and tnet (Opsahl 2009) in R version 3.2.2 (R Core Team 2011). To determine individual rank, ad libitum sampling was used to record the outcomes of all visible same-sex dyadic conflicts with no counter-aggression and used to calculate the dominance rank of each study animal using corrected normalised David's scores (de Vries et al. 2006). The proportion of scans in which an individual macaque was within proximity (within 10 m) of tourists was calculated over the entire study period, as was the rate of feeding or agonistic interactions. These physical interactions are likely to pose a greater risk of disease transmission than simple proximity or other interactions (e.g. looking at or photographing the macaque). Centrality in the network was compared with time spent within 10 m of tourists, the rate of interacting physically with tourists, rank and sex using multiple linear regression in R version 3.2.2 (R Core Team 2011). A permutation-based approach was used, to account for the non-independence of the data (Hanneman and Riddle 2005). Coefficients for each of the variables were compared with the results from 10,000 randomisations to determine significance. Randomisations were performed using the link reshuffling method in the tnet package (Opsahl 2009) for R (R Core Team 2011). This method randomly rewires links and their associated weights between group members. This introduces a high level of randomisation as it reshuffles both weights and the network topology while preserving the degree distribution (Opsahl 2009).

To explore the potential for each individual to infect other individuals in the network, a susceptible-infected-removed simulation model was employed. In these models, each individual in the network is at all times in one of three states: susceptible, infected or removed (Anderson and May 1991). One individual (patient zero) is selected to become infected at the start of the simulation, while all other individuals start as susceptible. The pattern of disease spread across the network depends on the basic reproductive number (R0), which varies with the relative infectiousness and recovery rate of the disease. Here, R0 was defined as the average number of secondary infections caused by one primary infection in a completely naïve population, following the methodology of Rushmore et al. (2014). Four different values of R0 were selected (0.7, 1.5, 3 and 10) to represent diseases with different levels of contagiousness, based on the human disease literature (Rushmore et al. 2014). We then calculated a rate of infection and a rate of removal (i.e. recovery), using the individual R0 values and the transmission probabilities between individuals in the network (see Rushmore et al. 2014 for full details). The simulation was run 10,000 times with each individual in the network as patient zero (i.e. a total of 170,000 simulations were therefore run), for each of four different values of R0 (0.7, 1.5, 3 and 10). At each step, individuals become infected or recover, based on the infection and recovery rate. After each simulation, the total size of the outbreak (the number of individuals who became infected during the epidemic) was calculated. This was averaged over the 10,000 simulations to give the mean size of outbreak caused when each individual macaque was patient zero. Multiple linear regression was used to investigate the relationships between the mean size of the outbreak caused by each individual, under each R0 value, and rank, sex and the time that they spent in proximity to, or interacting physically with, tourists.

### Impacts of Illness on Macaque Behaviour

Over the whole study period, the macaques were seen occasionally coughing and sneezing. However, there were three periods when a high proportion of individuals was seen coughing heavily and sneezing numerous times per day: from 6th to 20th March, from 23rd June to 9th July and from 22nd to 29th December 2012. The time spent in proximity with tourists and the rate of interacting (including both feeding and aggression) with tourists was calculated for each macaque observed with respiratory symptoms during each of these illness periods and compared with the rate during control periods (respectively, 9th February to the 23rd February, 24th May to the 10th June and 30th November to the 6th December). These control periods were of an equivalent length of time to each of the three illness periods, each ending 2 weeks before the first macaque showed symptoms of infection. This 2-week lag was to ensure that the control period ended before any of the macaques became infected as most respiratory infections take less than two weeks before showing symptoms (Lessler et al. 2009). In addition, it was important to ensure that the control periods were taken at a similar time of year to the illness periods, to minimise the effects of potential confounding variables such as tourist pressure and seasonal effects on behaviour. For each illness period and its associated control, interaction rates were compared using paired-sample *t* tests in R (R Core Team 2011). The same test was used to check that tourist numbers present in the area—i.e. within 100 m of the core of the macaque group (Maréchal et al. 2011)—did not differ between illness and control periods as, if they did, this could potentially affect interaction rates.

To test the validity of the use of association networks for disease modelling, association network metrics were compared between networks based on the illness periods and those based on the control periods. First, in order to ensure that the networks created over such short periods of time were reliable, the correlations between networks from the control period and the overall study period network were explored using the QAP in UCINET (Borgatti et al. 2002). Next, the means of individuals’ weighted degree centrality, weighted betweenness centrality, weighted eigenvector centrality and of network density were compared between the networks from the illness periods and their respective controls, using permutation-based paired-sample *t* tests with 10,000 permutations in R (for measures of centrality) (R Core Team 2011) and using the compare densities function in UCINET (for density) (Borgatti et al. 2002). The density of the network represents the number of connections present in the whole network in relation to the total number of possible connections; thus, a network with a high density is highly interconnected (Croft et al. 2008). Comparing these measures will therefore give an indication of any changes in the overall connectedness of the network, which is crucial for the spread of disease (Wey et al. 2008).

Finally, for each macaque observed coughing or sneezing, activity budgets (time spent in aggressive behaviour, feeding, resting, grooming, travelling and vigilance) were compared between the illness and control periods using Wilcoxon-matched pair tests in SPSS v.21 (© IBM Corp., 2012). To control for type I error rate, we used Bonferroni correction with α < 0.05 divided by the number of tests (*n* = 6). Therefore, after Bonferroni correction, significance level was α < 0.008.

## Results

**Fig. 1:**
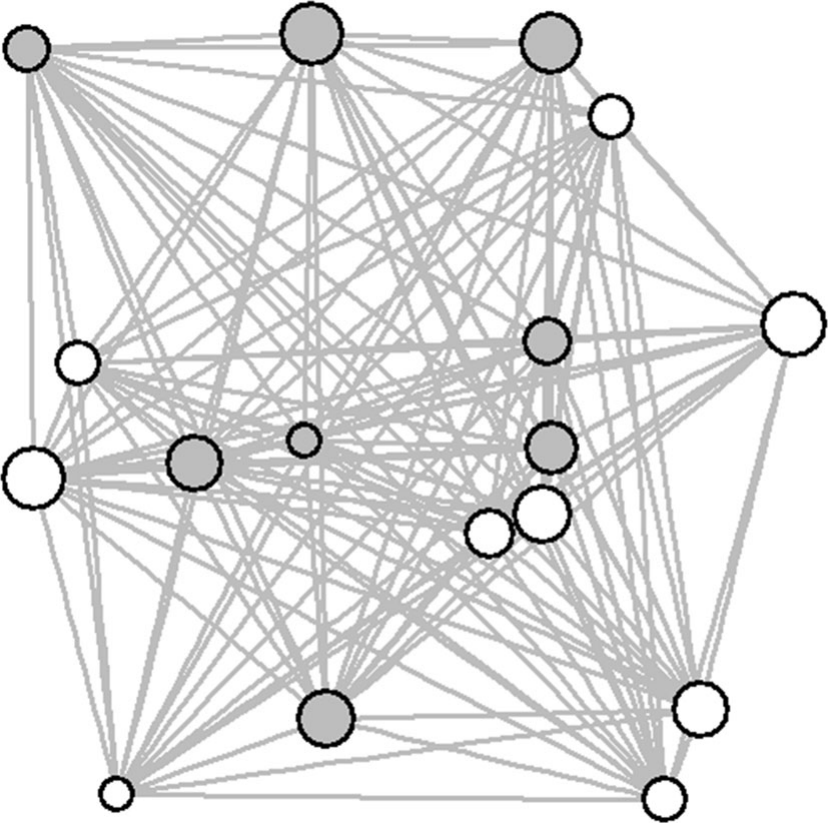
The association network based on nearest neighbours within 10 m. The size of the nodes reflects the interaction rate with tourists. Grey-filled nodes are males, and white are females.

A sociogram of the network is displayed in Fig. 1. On average, macaques were within 10 m of tourists in 49.0% of scans (range across study animals 35.0–57.0%) and interacted with tourists in 4.6% of scans (range 2.4–7.3%). The mean weighted degree of the macaque network based on nearest neighbours within 10 m was 0.442 (range 0.294–0.561). Mean weighted betweenness was 6.324 (range 0–24), and mean weighted eigenvector centrality was 0.764 (0.461–1). The density of the network was 1.0, meaning that all individuals were connected to all others in the network. Seasonal networks were shown to be highly correlated with the overall network, indicating that the network was relatively stable over time and therefore an accurate representation of the overall social structure of the group (Table 1). In addition, the 10 m nearest-neighbour network was highly correlated with the network based on nearest neighbours within 1 m (QAP matrix correlation = 0.765, *P* = 0.0002).

**Table 1:** Correlations Between Seasonal Networks and the Overall Network Based on the Whole Study Period, Calculated Using the Quadratic Assignment Procedure.

	Winter 1	Spring	Summer	Autumn	Winter 2
*R*	0.531	0.921	0.924	0.707	0.449
*P*	**<** **0.001**	**<** **0.001**	**<** **0.001**	**<** **0.001**	**<** **0.001**

Significant values are highlighted in bold.

### Are Macaques’ Rank, Sex or Level of Social Integration Related to Probability of Being in Proximity to, or Interacting with, Tourists?

There was no relationship between the proportion of scans in which individuals were in proximity to tourists and their rank, sex, weighted degree centrality or weighted betweenness centrality in the network (Table 2). However, there was a significant negative relationship between proportion of scans in proximity to tourists and eigenvector centrality (Table 2). There was no relationship between the rate of interacting physically with tourists (i.e. feeding or agonistic interactions) and sex, weighted degree centrality or weighted betweenness centrality (Table 3). The rate of physically interacting with tourists was significant positively related to weighted eigenvector centrality and significantly negatively related to rank (i.e. more dominant animals had lower rates of such interactions) (Table 3).

**Table 2:** Results of Permutation-Based Linear Regression (Based on 10,000 Permutations) to Explore the Relationship Between the Proportion of Time Spent in Proximity to Tourists and the Rank, Sex, Weighted Degree, Betweenness and Eigenvector Centrality of the Macaques (*n* = 17).

Variable	Coefficient	Range of coefficients	Standard error	*P*
Rank	− 0.001	− 0.041 to 0.042	< 0.001	0.084
Sex	0.081	− 0.010 to 0.179	< 0.001	0.471
Weighted degree centrality	0.602	− 3.031 to 2.134	0.006	0.134
Weighted betweenness centrality	− 0.004	− 0.039 to 0.038	< 0.001	0.688
Weighed eigenvector centrality	− 0.174	− 0.472 to 0.495	0.001	**0.018**

Significant values are highlighted in bold.

**Table 3:** Results of Permutation-Based Linear Regression (Based on 10,000 Permutations) to Explore the Relationship Between the Rate of Interacting with Tourists and the Rank, Sex, Weighted Degree, Betweenness and Eigenvector Centrality of the Macaques (*n* = 17).

Variable	Coefficient	Range of coefficients	Standard error	*P*
Rank	− 0.001	− 0.004 to 0.007	<0.001	**0.011**
Sex	0.014	0.002 to 0.028	<0.001	0.783
Weighted degree centrality	− 0.123	− 0.376 to 0.441	0.001	0.073
Weighted betweenness centrality	− 0.001	− 0.006 to 0.005	<0.001	0.633
Weighed eigenvector centrality	0.084	− 0.057 to 0.074	<0.001	**<0.001**

Significant values are highlighted in bold.

### Is the Size of Simulated Disease Outbreak Related to the Initially Infected Macaque’s Rank, Sex, Time in Proximity to, or Rate of Interaction with, Tourists?

**Fig. 2:**
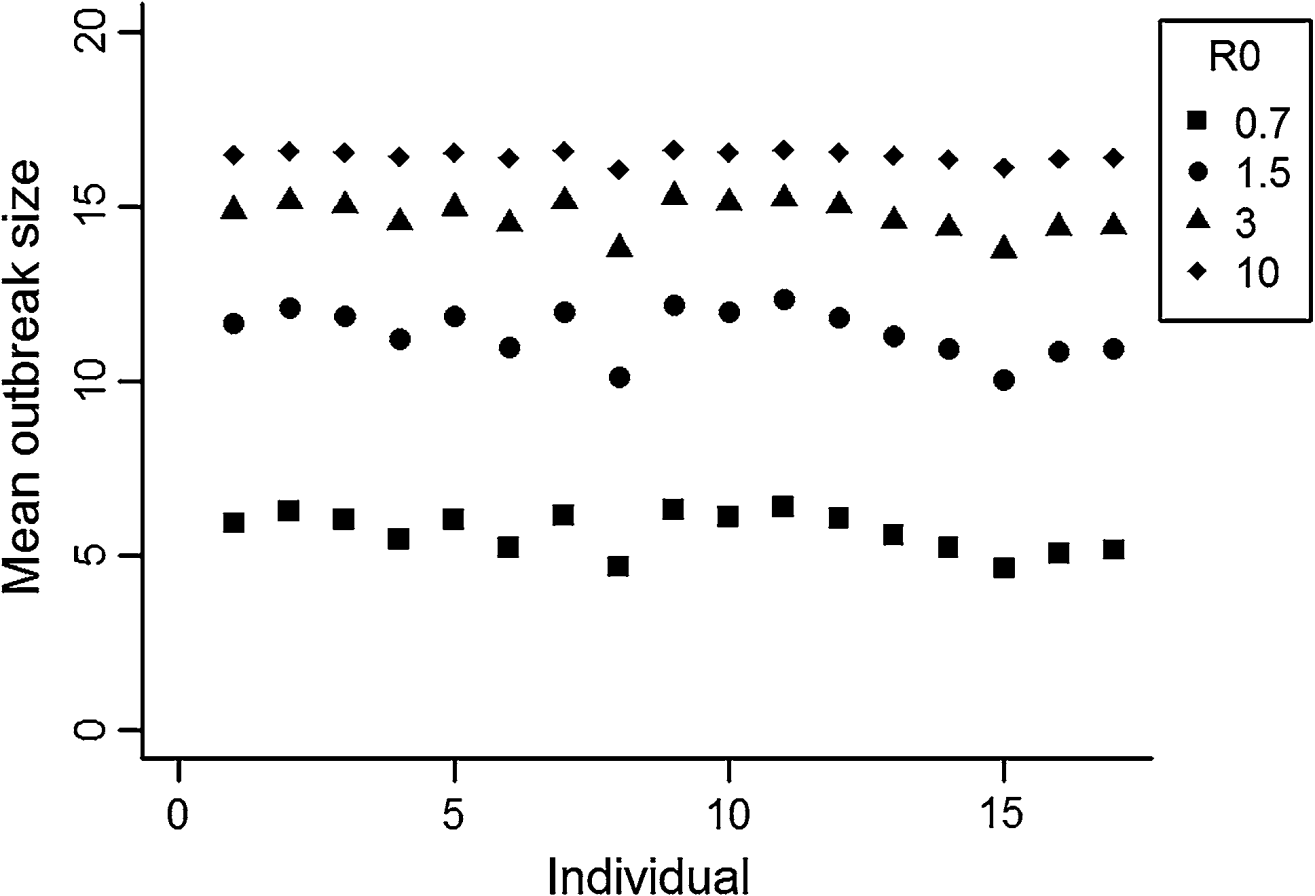
Mean size of the predicted outbreak with each individual (*n* = 17) as patient zero (over 10,000 simulations) at four different values of basic reproductive number, R0 (0.7, 1.5, 3 and 10). Individuals are arranged in rank order (1–8 are males, and 9–17 are females), in order of descending rank.

For all values of R0, there was very little variation in the size of outbreak caused by different individuals (Fig. 2). No significant relationships were found for any R0 value between the mean size of the simulated disease outbreak caused by an individual and their sex, the proportion of time they spent in proximity to, or their rate of physical interactions with, tourists (Table 4). There was a significant positive relationship between rank and the mean size of the simulated outbreak (i.e. more dominant animals caused larger outbreaks) for all four values of R0 (Table 4).

**Table 4:** Results of Linear Regression to Explore the Relationship Between the Mean Outbreak Size and the Proportion of Time Spent in Proximity to Tourists, the Rate of Interacting with Tourists, Rank and Sex at a Range of R0 Values (*n* = 17).

R0	Behaviour	Coefficient	Standard error	P
0.7	Proximity	0.121	2.667	0.964
0.7	Interactions	3.887	17.693	0.830
0.7	Rank	0.168	0.046	**0.003**
0.7	Sex	0.119	0.276	0.674
1.5	Proximity	− 0.037	3.433	0.992
1.5	Interactions	5.745	22.776	0.805
1.5	Rank	0.193	0.059	**0.007**
1.5	Sex	0.115	0.356	0.751
3	Proximity	− 0.086	2.403	0.972
3	Interactions	3.219	15.940	0.843
3	Rank	0.128	0.041	**0.009**
3	Sex	0.087	0.249	0.732
10	Proximity	− 0.110	0.874	0.902
10	Interactions	2.375	5.801	0.689
10	Rank	0.039	0.015	**0.024**
10	Sex	0.004	0.091	0.959

Significant values are highlighted in bold.

### Does Illness Change Macaques’ Behaviour Towards Tourists?

During the illness periods, ill macaques spent an average of 19.7% of scans (ranging from 0.0 to 32.2%) in proximity to tourists (within 10 m) and interacted with tourists on average in 4.1% of scans (ranging from 0.0 to 10.4%). There were no significant differences in time spent in proximity to tourists between the illness periods (IPs) and their associated control periods (CPs) (paired-sample *t* tests: IP1 mean 22.2% of scans; CP1 mean 25.4% of scans; *t* = −1.133, *df* = 16, *P* = 0.274; IP2 mean 23.6% of scans; CP2 mean 21.1% of scans; *t* = 1.171, *df* = 12, *P* = 0.265; IP3 mean 9.3% of scans; CP3 mean 14.8% of scans; *t* = −1.456, *df* = 8, *P* = 0.184). Ill macaques interacted significantly less with tourists during the first illness period than in the associated control period (IP1 mean 3.5% of scans; CP1 mean 6.6% of scans; paired-sample *t* test: *t* = −2.920, *df* = 16, *P* = 0.010), but there were no significant differences in interaction rates with tourists between the other two illness periods and their control periods (paired-sample *t* tests: IP2 mean 4.8% of scans; CP2 mean 4.8% of scans; *t* = −0.116, *df* = 12, *P* = 0.910; IP3 mean 4.2% of scans; CP3 mean 5.6% of scans; *t* = −0.975, *df* = 8, p = 0.358). The mean numbers of tourists per scan per day did not differ significantly between illness and control periods (paired-sample *t* tests: IP1 and CP1 mean, respectively, 9.8 and 7.0 tourists per scan per day; *t* = 0.768, *df* = 10, *P* = 0.460; IP2 and CP2 mean, respectively, 16.5 and 14.6 tourists per scan per day; *t* = −0.527, *df* = 12, *P* = 0.608; IP3 and CP3 mean, respectively, 7.3 and 11.1 tourists per scan per day; *t* = 1.011, *df* = 4, *P* = 0.369).

### Do Macaque Association Networks and Behavioural Activities Change in Response to Outbreaks of Illness?

Networks based on the control periods were highly and significantly positively correlated with the overall study period network, suggesting that the network is stable over time and that it was possible to capture a reliable network over these short time periods (Table 5).

**Table 5:** Results of Correlations Between Networks Based on the Control Periods and the Overall Network Based on the Whole Study Period, Calculated Using the Quadratic Assignment Procedure.

	Control 1	Control 2	Control 3
Overall network			
*R*	0.573	0.708	0.296
*P*	**<** **0.001**	**<** **0.001**	**<** **0.01**

Significant values are highlighted in bold.

Networks did not differ significantly between the illness and the control periods with respect to density or any of the three measures of individual centrality, suggesting that macaques do not change the number or strength of their social interactions when infected with a respiratory illness (Table 6).

**Table 6:** Results of Comparisons (Paired-Sample *t* Tests with 10,000 Permutations) of Mean Weighted Degree Centrality, Mean Weighted Betweenness Centrality, Mean Weighted Eigenvector Centrality and Density Between Networks Based on Data from the Three Illness Outbreaks and Their Associated Control Periods (*n* = 17).

	Illness period	Control period	*t*	Standard error	*P*
Mean weighted degree centrality	0.441	0.446	− 0.256	0.011	0.853
Mean weighted betweenness centrality	10.382	10.206	0.094	0.010	0.936
Mean weighted eigenvector centrality	0.736	0.724	0.367	0.011	0.846
Density	0.827	0.860	− 0.172	0.002	0.222
Mean weighted degree centrality	0.451	0.455	0.255	0.012	0.896
Mean weighted betweenness centrality	7.147	9.294	1.569	0.010	0.259
Mean weighted eigenvector centrality	0.747	0.666	− 2.724	0.011	0.240
Density	0.897	0.904	− 0.175	0.003	0.418
Mean weighted degree centrality	0.398	0.343	1.207	0.011	0.313
Mean weighted betweenness centrality	11.618	8.235	1.575	0.010	0.119
Mean weighted eigenvector centrality	0.539	0.497	0.557	0.012	0.638
Density	0.471	0.397	− 0.784	0.004	0.087

The comparisons of activity budgets between illness and control periods (Table 7) indicated that macaques were more vigilant during illness period 1 (IP1 and CP1 means, respectively, 1.2 and 0.0% of scans), while there was no significant difference in vigilance between illness and control periods for periods 2 or 3. Aggressive behaviour, feeding, grooming, travelling and resting were not significantly different between any pairs of illness and control periods.

**Table 7:** Results of Comparisons (Wilcoxon-Matched Pairs Test) of Animals’ Activity Budgets Between Each Period of Illness and Its Associated Control Period.

	Aggressive behaviour	Feeding	Resting	Grooming	Travelling	Vigilance
Period 1 (*n* = 17)						
*Z*	− 1.098	− 1.018	− 1.538	− 0.970	− 0.166	− 2.803
*P*	0.272	0.309	0.124	0.332	0.868	**0.005**
Period 2 (*n* = 13)						
*Z*	− 0.765	− 0.035	− 0.804	− 2.132	− 2.551	− 2.402
*P*	0.444	0.972	0.422	0.033	0.011	0.016
Period 3 (*n* = 9)						
*Z*	− 0.314	− 1.599	− 0.237	− 0.652	− 1.599	− 0.338
*P*	0.753	0.110	0.813	0.515	0.110	0.735

For each period, *n* indicates the number of animals that were ill and hence is the sample size for the comparison. Significant values after Bonferroni correction was applied (corrected α = 0.008) are highlighted in bold.

## Discussion

The Barbary macaques at this tourist site in Morocco spent a large proportion of their time in sufficiently close proximity to tourists for aerosol transmission of disease, and also interacted with tourists regularly, even when showing signs of infectious diseases. There are no regulations at the study site to restrict such interactions, and tourists regularly feed the monkeys and are aggressive towards them (Maréchal et al. 2011, 2016). These types of interactions clearly have high risk of anthropozoonotic disease transmission. The probability of exchange of fluids between tourists and primates is particularly high when tourists give food to monkeys (Honess et al. 2006), or during aggressive interactions, especially when physical contact occurs. Potential fluid exchange during provisioning has been observed at the study site, for example when tourists crack peanut shells in their mouth before giving them to the monkeys (Maréchal et al. 2016).

Individual macaques varied in their proximity to, and rate of interaction with, tourists. When controlling for all other variables, being more socially integrated (as measured by eigenvector centrality) was associated with spending less time in proximity to tourists, but also with more time interacting with them. Rank was significantly negatively related to the rate of interacting with tourists, indicating that higher-ranked individuals interacted less than lower-ranked monkeys and thus may be less prone to becoming infected. Other measures of centrality, and sex, were not related to the time spent in proximity to, or rate of interacting with, tourists. Overall, these results suggest that there is no clear and consistent link between rank, sex or centrality and behaviour towards tourists; these factors therefore would not appear to be particularly useful in informing potential disease prevention strategies, such as targeted vaccination.

In addition to variation in behaviour towards tourists, depending on the association network structure, there may also be variation in the ability of different individuals to spread disease within the group. If those that are at high risk of becoming infected are also able to cause large outbreaks, this would be particularly concerning. However, the simulations presented here indicated that there was little variation in the size of potential outbreaks, depending on which individual became infected first. The same pattern was found when modelling the spread of diseases varying in contagiousness. This suggests that there are no potential ‘super-spreaders’ in this group of macaques. This is similar to findings from chimpanzees (Carne et al. 2013, 2014) but contrasts with those from studies of a number of other (non-primate) mammal species where ‘super-spreaders’ have been identified (Lusseau 2003; Manno 2008; Clay et al. 2009), as well as with data from other primate species (Griffin and Nunn 2012; Nunn 2012). Among Japanese macaques, for example, simulations have indicated that more central individuals transmit infections in a shorter amount of time and to more subjects than less central animals (Romano et al. 2016). There is also evidence that central individuals have greater parasite loads in Japanese macaques (MacIntosh et al. 2012) and red-capped mangabeys (Friant et al. 2016), indicating that these individuals may also be at greater risk of contracting a disease. This variation in results across species may be related to the species-specific degree of tolerance/despotism. More tolerant species have been found to have more highly connected networks (Pasquaretta et al. 2014), and so it is possible that the observed differences in the degree of network connectivity are linked to variation in levels of tolerance and despotism among the species that have been studied to date.

Importantly, there was also no significant relationship between the mean size of the simulated outbreaks caused and the proportion of time that individuals spent with tourists, their interaction rate with them, or their sex. There was, however, a significant positive relationship with rank, indicating that infection of higher-ranked individuals was associated with greater outbreak sizes. This suggests that rank is the most important variable of those tested in determining the potential for an individual to spread disease within the group. However, it should be highlighted that the absolute differences in outbreak size between individuals predicted here were minimal, and so while the pattern may be statistically significant, in practical terms the difference is very small. It is also interesting to note that animals with higher rank were less likely to interact with tourists, so while they may have a slightly greater ability to spread disease within the group, they are less likely to contract an initial infection from tourists.

Although from a conservation perspective it is important to look at the potential for disease transmission from tourists to primates, from a one health perspective (i.e. an initiative to unite human, animal and environmental health) it is also crucial to consider the potential for disease to spread in the opposite direction (Zinsstag et al. 2011). The behaviour of the macaques towards tourists while infected was analysed here to try to quantify the level of this threat. Although interaction rates with tourists were lower during the first illness period than during the associated control period, in the other two illness periods there were no significant differences in the rates of interactions with tourists between the illness periods and their respective controls. This suggests that there is a risk of tourists contracting an infection from the macaques. During the first illness period, all 17 of the studied macaques appeared to become infected, compared to only 13 and 9 in illness periods 2 and 3, respectively. It is possible that the illness in period 1 was a more virulent one that led to changes in macaques’ behaviour towards tourists. The tourists seemed to have little knowledge or understanding of the potential risks of disease transmission between themselves and the macaques (Maréchal 2015); a similar situation has been reported at Sepilok Orangutan Rehabilitation Centre in Borneo, where tourists did not seem to realise the potential disease transmission risks for the orangutans they came to visit (Muehlenbein et al. 2010). It would be valuable in the future to assess more fully the level of understanding among the tourists of the conservation and personal health issues associated with close interactions with wildlife, including feeding wildlife.

Our comparison of behavioural activities and network metrics between the illness periods and their respective controls indicated there were no significant differences in network density or centrality, nor in rates of aggressive behaviour, feeding, grooming, travelling and resting between these times. Interestingly, while in the first illness period the macaques reduced their interactions with tourists, there were no significant differences in network metrics between the illness and the control period. This suggests that even though the monkeys may change their behaviour towards tourists, their association network stays relatively constant and can still be seen as an accurate representation of their social structure. Although the networks were based on a short period of time, the networks from the control period were highly correlated with the overall study period network, providing support for their reliability. Overall, these results attest to the utility of using networks derived from shorter timescales in generalising to longer timescales when using modelling approaches for the simulation of disease spread. However, it is important to note that all of the actual outbreaks analysed here were of mild respiratory diseases. While these types of diseases can have dramatic impacts on primate survival (Koendgen et al. 2008) and so are an important concern, it is possible that more virulent diseases would affect the structure of the association networks and thus the observed patterns of transmission.

Although for most of the measures analysed, there was little evidence that the macaques changed their behaviour in response to disease, there was some indication that these animals were more vigilant during the first period of illness (potentially the most virulent). In a range of species, it has been shown that infected individuals are at higher risk of predation than healthy animals (Scott 1988; Hudson et al. 1992; Johnson et al. 2006), perhaps because they are weaker and less able to escape. It is therefore possible that the macaques are at a higher risk of predation when infected with more virulent diseases and that they increase their levels of vigilance in response.

### Limitations of the Study

It is important to note that this study has some limitations that must be considered when interpreting the results. Nearest-neighbour data were used to construct the networks, and this led to a completely connected network, with little social differentiation. With all individuals connected, the impact of the identity of patient zero in the disease simulations is reduced. It would be interesting to compare these results with those for a network based on grooming relationships, to see whether findings differ. Although time spent grooming and time spent as a nearest neighbour would be expected to be correlated, grooming networks in primates are not usually completely connected (Lehmann and Ross 2011; Brent et al. 2013; Wikberg et al. 2015), as was found here for the nearest-neighbour network. Additionally, using nearest-neighbour data means that some important relationships may not be represented in the data. For example, if an individual was within 3 m of the focal animal, but there was another individual closer to the focal animal, the former individual was not recorded as nearest-neighbour under our procedure. However, if the individual was within 9 m of the focal animal and no other animal was closer to the focal animal, it was recorded as the nearest neighbour. Thus, using these data to calculate association networks may miss some relationships. Furthermore, it is possible that social relationships of more central individuals are being underestimated relative to peripheral individuals, as at any one time a central animal may have multiple neighbours, but only one is recorded. Nevertheless, when collected over a long period of time, nearest-neighbour data would still be expected to provide an accurate representation of the overall structure of the network.

The current study did not include infants, juveniles, sub-adults or young adults in the association network. Such animals might play an important role in the contraction and dissemination of infectious diseases (Nunn and Altizer 2006). For example, adult females, infants and juveniles spend a lot of time in close proximity or directly interacting, often at the core centre of the group, and so are at higher risk of disease transmission (Rushmore et al. 2013). Moreover, Barbary macaque infants play an important role in social behaviours of adult males (Paul et al. 1996) as well as females, increasing the number of potential partners infected. Younger individuals, as well as older ones, might also have lower immunity against infectious diseases than healthy adults and thus might be more susceptible to disease (Nunn and Altizer 2006). Further research should consider including all animals in the social group, to provide a more accurate estimate of the potential disease transmission risks with tourists.

Currently, there is no information available on precise disease parameters in Barbary macaques and so it is not possible to simulate the spread of specific disease-causing agents. However, it is reasonable to assume these animals will be susceptible to many of the same diseases as humans, but that the level of morbidity or mortality may be higher among the macaques as a result of a lack of prior immunity/exposure to these human pathogens (Muehlenbein and Wallis 2014). Indeed, great apes have been found to be highly susceptible to diseases that cause only relatively minor sickness in humans, such as influenza (Wallis and Lee 1999). Thus, disease spread from tourists could have serious negative effects for the monkeys. Here we did not record the number of tourists that were displaying signs of infection, but this would be an interesting avenue for future research. In addition, it is important that more information is collected on disease both in groups visited by tourists and those living in undisturbed areas, to parameterise more specific models, and to further elucidate the potential impact of tourists on the monkeys.

### Implications for Management

Overall, the findings of this paper, and in particular the evidence that macaques spend a high proportion of their time in close proximity to, and interacting with, tourists, even when showing signs of illness, highlight the potential risks at our field site of disease transmission from tourists to Barbary macaques and vice versa. Although individuals with higher rank had a significantly greater ability to spread disease within the group, in absolute terms, the differences between individuals were minimal. As such, there do not appear to be ‘super-spreaders’ in the group, meaning that targeting particular individual macaques for preventative measures, such as vaccinations, would be unlikely to be successful. This means that more general preventative measures, such as tourist education and keeping a safe distance between tourists and macaques, are more likely to be successful in mitigating any risks of spread, by attempting to reduce the contact rate between tourists and macaques. Comprehensive guidelines to mitigate such risks should therefore not be restricted to great ape tourism (Macfie and Williamson 2010; Gilardi et al. 2015), but rather extended to—and most importantly enforced in—tourism related to the other primates and wildlife more broadly.
